# Innovative motor and cognitive dual-task approaches combining upper and lower limbs may improve dementia early detection

**DOI:** 10.1038/s41598-021-86579-3

**Published:** 2021-04-02

**Authors:** Gianmaria Mancioppi, Laura Fiorini, Erika Rovini, Radia Zeghari, Auriane Gros, Valeria Manera, Philippe Robert, Filippo Cavallo

**Affiliations:** 1 The BioRobotics Institute, Scuola Superiore Sant’Anna, Viale Rinaldo Piaggio 34, Pontedera, 56025 Pisa, Italy; 2 The Department of Excellence in Robotics & AI, Scuola Superiore Sant’Anna, piazza Martiri della Libertà 33, 56127 Pisa, Italy; 3 The Department of Industrial Engineering, University of Florence, via Santa Marta 3, 50139 Florence, Italy; 4 The CoBTeK, Université Cote d’Azur (UCA), 10 Rue Molière, 06100 Nice, France

**Keywords:** Cognitive ageing, Cognitive neuroscience, Motor control, Biomarkers, Neurological disorders, Neurological disorders, Biomedical engineering

## Abstract

Motor and Cognitive Dual-Task (MCDT) represents an innovative chance to assess Mild Cognitive Impairment (MCI). We compare two novel MCDTs, fore-finger tapping (FTAP), toe-tapping (TTHP), to gold standards for cognitive screening (Mini-Mental State Examination—MMSE), and to a well-established MCDT (GAIT). We administered the aforementioned MCDTs to 44 subjects (MCIs and controls). Motor parameters were extracted, and correlations with MMSE investigated. Logistic regression models were built, and AUC areas computed. Spearman’s correlation demonstrated that FTAP and TTHP significantly correlate with MMSE, at each cognitive load. AUC areas computed report similar (FTAP, 0.87), and even higher (TTHP, 0.97) capability to identify MCIs, if compared to GAIT (0.92). We investigated the use of novel MCDT approaches to assess MCI, aiming to enrich the clinical repertoire with objective and non-invasive tools. Our protocol shows good correlations with MMSE, and reaches high performances in identifying MCI, adopting simpler exercises.

## Introduction

Due to the rapid global aging, people affected by dementia will triple worldwide in the next 30 years^1,2^. The circumstances are exacerbated by the fact that, for the majority of the cases, no effective curative treatment is available so far. That justifies the increasing efforts to identify reliable predictors of the disease^3^. Besides, the scientific community advocates the identification of valid, inexpensive, and non-invasive markers, that may improve the early detection of these conditions^4,5^. In this framework, Mild Cognitive Impairment (MCI), a 10-fold risk condition of progressing to dementia^6^, represents a crucial target for clinical research. The early detection of MCI, and therefore timely interventions, implies benefits for those patients. For instance, reversible conditions can be identified and treated (e.g. metabolic and endocrine diseases, mood and sleep disorders, and iatrogenicity), as well as specific lifestyle habits that may slow down or even prevent further cognitive decline can be implemented for unrecoverable ones^7^. Dual-Task approaches, sensitive tests for MCI, represent a thriving field for the application of Information and Communications Technologies (ICT) on clinical practice^8,9^, and recently has been incorporated into a novel approach for the assessment of the cognitive status: the Motor and Cognitive Dual-Task Approach (MCDT). Nowadays, human motor activity is not merely considered as an automatic behavior, but rather the outcome of a widespread and complex network^10,11^. Gait abnormalities, barely detectable at the naked eye, are exacerbated and unmasked using appropriate protocols. The MCDT is a “brain-stress test”^12^ developed to evaluate the functioning of the motor-cognitive interface. The rationale behind MCDT is that cognition is embodied. Therefore, movements would require cognitive supplies^13^. MCDT encompasses motor tasks (the most adopted is free walking task) and cognitive tasks (e.g. counting backwards or less commonly naming animals) as competitive task. The idea is that concurrent tasks would compete for cognitive resources. Therefore, cognitive load should consume the subject’s cognitive reserve, subtracting it to the motor-control mechanisms and revealing abnormalities^10,12,14^. Different combinations of motor tasks and cognitive exercises are now encouraged to be explored^15^, and groundbreaking experiments have been carried on in the last years; for instance the Toosizadeh et al. works on association of upper-extremity function (UEF) under dual-task (DT) condition and cognitive status^16,17^. In conclusion, even if the core MCI’s hallmark is the cognitive impairment, several studies found that also motor dysfunctions can occur^12,18^. The use of motor parameters as reliable information on cognitive subjects’ status represents an emerging research point and notably, it has been proven to be advantageous in identifying several typologies of MCI, both regarding the etiology (MCDT paradigms successfully have been adopted as well in Parkinson’s disease^19^ as in Alzheimer’s disease^20,21^), or the neurocognitive domains involved. In fact, such an approach has proven to be appropriate both with amnestic or non-amnestic-MCI^22^ and even comparing amnestic-MCI-single domain vs amnesitc-MCI-multiple domain^14^. That is due to its flexibility, which allows the clinician to combine different motor and cognitive tasks, generating varied and tailored protocols. The study of the motor-cognitive interface through MCDT protocols is enriching the repertoire of neuropsychological tests for the early dementia screening offering to the clinicians new reliable solutions. It represents a flourishing field of study with really pragmatic outcomes. In this study, we present two novel solutions for MCDT, particularly the use of two tapping tasks (fore-finger tapping and toe-tapping heel pin, respectively named: FTAP and TTHP). Technological solutions for MCDT so far mostly rely on non-portable systems and walking DT approaches. We propose a new wearable system (SensHand and SensFoot), that would allow the clinicians to perform easier and more convenient tasks. We aim to verify the existence of correlations between the proposed tasks and the gold standard for cognitive assessment (the Mini-Mental State Examination, MMSE). Then, we would compare FTAP and TTHP to the gold standard for MCDT, which is represented by the mainstream walking task (here named GAIT). To achieve these goals, we adopt a two-step procedure. Firstly, we performed Spearman’s correlations between the motor parameters extracted by each exercise (namely, FTAP, TTHP, and GAIT) and the MMSE score, to observe whether FTAP and TTHP could be considered as an approximation of global cognitive status. Then, we adopt logistic regressions models to distinguish MCI and CNA subjects. We included receiver operating characteristic (ROC) curves to display models’ performance.

We hypothesize (1) to observe statistically significant associations between MCDT parameters and the MMSE scores, which would mean that FTAP and TTHP performances would account for at least a part of subjects’ global cognitive status; (2) that FTAP and TTHP might be comparable in terms of specificity and sensitivity with MCDT gold standard, namely GAIT. That last point, the real core of this pilot study, would mean that it is possible to implement more accessible and straightforward MCDT solutions for MCI cognitive screening.

## Materials and methods

### Participants

Seventy older adults were recruited and assessed, as detailed in the supplementary material, within this study. Forty-four (67%) of them have fulfilled all the requirements and were able to complete the full physical protocol. Seventeen were subject clinically evaluated as affected by Mild Neurocognitive Disorder^23^, while 27 were considered Cognitively Normal Adults (CNA). We recruited them at the Memory Center (CMRR) of Nice University Hospitals (CHU of Nice, France) and at the CoBTeK research lab of the Université Cote d’Azur, in the context of Marco-Sens multi-centric research protocol. Experimental subjects, diagnosed as Mild Neurocognitive Disorder (a diagnostic category that stems from experiments on MCI^24^) had in their records, at least: blood tests, encephalic MRI, and a neuropsychological assessment according to the French health authority recommendations. Participants (both MCIs and CNA) were not included if they had sensory or motor impairments interfering with the protocol completion, present moderate to severe cognitive impairment attested by standard neuropsychological screening test (MMSE), or they were participating in any cognitive stimulation/training program. Referring to the 27 cognitively intact subjects, they were recruited at the CMRR among the patients’ caregivers, CMRR personnel, and the CMRR network. A neuropsychological screening (including the MMSE) was performed to ascertain the absence of any cognitive decline. The study was performed in compliance with the Declaration of Helsinki and was approved by the National Ethical Committee—Comité de Protection des Personnes—on 15/04/2019 ($\hbox {N}^{\circ }$ ID RCB: 2019–A00342-55). All participants received detailed written explanations on the study aims and procedures and provided their informed written consent before taking part in the study.

#### Clinical assessment

Clinical measurements reported in this study included the MMSE, which is considered as the gold standard tool for cognitive screening of cortical dementia^25^. Notably, the Supplementary Material encompasses the whole neuropsychological battery we adopted for the clinical characterization of our sample. tests used and the Frailty Index, based on the Fried frailty phenotype criteria^26^. Participants with MMSE score lower than 24 (adjusted with normative age and educational level) were identified as those with moderate to severe cognitive impairment^27^, and, therefore, excluded from our study. The Frailty Index, which regards unintentional weight loss, self reported exhaustion, weakness (grip strength), slow gait speed (6 meters) and self-reported low physical activity, was used to assess frailty^26^. Individuals with three or more positive Fried criteria were considered frail, one or two were considered pre-frail, and those none non-frail. For the sake of completeness the full list of the neuropsychological tests adopted is encompassed in the Supplementary Materials, attached to this work.

### Instrumentation

**Figure 1:**
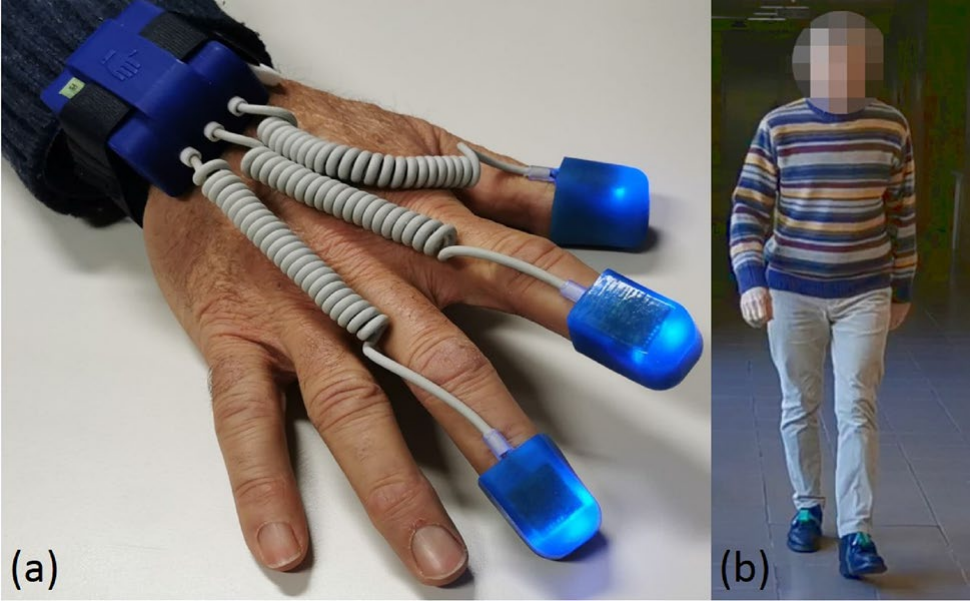
(**a**) SensHand system; (**b**) SensFoot system.

For our research, we used a wearable system, based on microelectromechanical sensors (MEMS), composed by the SensHand and the SensFoot devices. SensFoot is a single inertial measurement unit (IMU) integrated into the iNEMO-M1 board (STMicroelectronics, Italy) to measure the lower limb motor performances. It encompasses a three-axis gyroscope L3G4200D for measuring angular velocities, a six-axis geomagnetic module LSM303DLHC for acquiring accelerations, and a ARM-based 32-bit microcontroller STM32F103RE (STMicroelectronics, Italy) (see Fig. 1). The device is fixed on the dorsum of the subjects’ foot with a Velcro strap to avoid movements between the foot and the device^28^. Whereas, SensHand was applied to assess the upper limb motor performances. It is composed of four customized IMU-based boards, with a coordination unit included in a bracelet and three finger units placed in as many ring packages on the distal phalanxes of the thumb, index and middle finger. The coordination unit communicates with the rings through spiral cables exploiting the Controller Area Network (CAN-bus) standard. Also, the bracelet unit synchronizes the data exchange between the nodes of the device. Inertial sensor signals are acquired at a frequency rate of 100 Hz from each unit of SensHand and SensFoot. Both the devices are integrated with Bluetooth modules for the wireless transmission of the acquired data to a remote personal computer, where a graphical user interface is used by clinicians for storing data and to offline analyze the motion parameters^29^ (see Fig. 1).

### MCDT protocol

Three MCDTs have been used in this study: GAIT, FTAP, and TTHP. GAIT represents the gold standard for MCDT assessment. Whereas, FTAP and TTHP extensively described in^30^, represent, for the sake of our knowledge, an innovation in MCDT protocols. Each exercise have been performed both in Single Task (ST) and DT condition. ST condition refers only to motor exercise, without any cognitive load (CL); the DT condition includes three cognitive load levels (CL1, CL2, CL3, respectively, counting backwards by 1, by 3, and by 7). Before starting the actual test, the protocol was explained to participants, and the ST and DT series were administered randomly. Furthermore, we ask the subjects to perform each task by their own pace, without any instruction about the task prioritization (physical task vs counting task). Notably, counting backwards has been selected as concurrent cognitive task, after a complete literature screening, which has evidenced that this task is the most commonly used for MCDT protocols^12,17,31^. Moreover, as stated by Toosizadeh et al., counting backwards involves working memory and, therefore is more directly related to executive functions, if compared to other tasks such as naming animals^16^. Besides, counting is a rhythmic task and may highly interfere with another rhythmic task that has a different frequency, such as walking or finger/toe-tapping^32^.

#### FTAP and TTHP

During the FTAP the subjects were asked maintain the hand fixed on the table for 3 s at the beginning (to acquire the baseline position) and then to tap their fore-finger at they own pace for 15 s, resting their fore-arm on the experimental desk, while they are wearing the SensHand on the dominant hand^33^ (See Fig. 1). Conversely, during the TTHP the subjects were asked to maintain their foot fully on the ground for 3 s (to acquire the baseline position) and then to tap their toe while the heel was pinned to the floor, for 15 s. The subjects had to wear the SensFoot on their dominant foot^33^ (See Fig. 1). Several outcome measures representing kinematics and kinetics of finger and toe-tapping were extracted (see in the Supplementary Material).

#### GAIT

GAIT was objectively assessed using SensFoot, worn on the subject’s dominant foot^33^. Gait was assessed by 10-meters straight walking test. First and last steps have not been considered for the parameters extraction, so to avoid acceleration and deceleration phenomena. As aforementioned, the subjects were asked to perform the task by their own pace, without any instruction about the task prioritization. Gait outcomes were computed as reported in the supplementary material.

#### Dual-task cost

As aforementioned, each of the above MCDT (FTAP, TTHP, and GAIT) parameters were measured within ST and DT conditions. To assess changes in individual’s performance from a ST to DT, “Dual-Task Cost” (DTC) was measured for each parameter, as percentage of change within the two conditions:

1 $$\begin{aligned} {DTC=\frac{Parameter_{DT}-Parameter_{ST}}{Parameter_{DT}} \times 100} \end{aligned}$$

### Signal processing and features extraction

Accelerations and angular velocities acquired by SensHand and SensFoot were off-line processed using Matlab2018b® (The MathWorks, Inc., Natick, MA, USA). The signals were filtered applying a fourth-order low-pass digital Butterworth filter with a 5 Hz cut-off frequency to eliminate high-frequency noise. Signal segmentation and motor parameters extraction were implemented through custom-developed algorithms. Particularly, as concern the FTAP and the TTHP, 11 features were extracted as described in^34^ by processing the angular velocity orthogonal to the movement and the acceleration vector; whereas, for GAIT, 16 parameters were extracted by analyzing the angular velocity orthogonal to the direction of the walking^28^. The complete list of the extracted features is reported in the supplementary materials. The set of 38 features was computed at each cognitive load, thus, for each exercise 4 datasets were computed, i.e. CL0, CL1, CL2, CL3. Additionally, for each feature the dual-task cost (DTC) was also calculated as the difference between the feature extracted in DT at CLi (where $\hbox {i}=1,$ 2 , 3) and the one extracted at CL0 (ST) divided by the one at CL0 (see Eq. 1). By the end of this process, totally 266 features have been computed and analysed.

### Statistical analysis

Preliminary descriptive analysis were performed on demographic, antropometric and clinical measures of CNA and MCI subjects. The Wilcoxon rank sum test for equal medians (equivalent to Mann-Whitney U test) has been used for continuous variables: age, stature, body mass, body mass index and MMSE. The $\chi ^{2}$ test, for testing independence of each dimension of occurrence, was used to assess categorical variables: educational level (primary, secondary, superior) and Fried Score (not frail, pre-frail, frail) (see Table 1). MCDT parameters (ST, DT, and DTC) were compared between the two groups using The Wilcoxon rank sum test. Notably, also the number of correct responses has been assessed. Whereas, correlations between MCDT parameters and MMSE score were assessed using Spearman’s correlation. Further, multiple logistic regression models were used to determine the association of MCDT parameters with subjects’ clinical status. These models (one model for each MCDT test at different CL) encompass MCDT parameters with statistically significant Wilcoxon in distinguishing between CNA and MCI ($\hbox {p}>0.05$). Notably, such parameters were considered as independent variables. Conversely, the cognitive status (CNA vs MCI) was considered as dependent variable. Importantly, age was considered as a covariate within those models. Receiver operating characteristic (ROC) curves were calculated, as well as respective sensitivities, specificities and areas under the curves (AUC). A summary of results is presented in the following sections. All analyses were performed using MATLAB (version 2018b).

**Table 1:** The $^{\dagger }$ symbol represents differences computed by the Mann–Whitney *U*-test.

Variable	CNA	MCI	p-value
Number (% of the group)	27 (61%)	17 (39%)	–
Female n (% of the group)	13 (48%)	13 (76%)	$0.063^{\ddagger }$
Age, year (IQR)	65 (13)	73 (12.75)	$<0.01\ ^{\dagger }$
Stature, cm (IQR)	168 (13)	162 (11.5)	$0.07\ ^{\dagger }$
BMI, $\hbox {kg}/\hbox {m}^{2}$ (IQR)	24.1 (4.9)	25.7 (6.5)	$<0.01\ ^{\dagger }$
**Education level**			$0.130^{\ddagger }$
Primary, n (%)	1 (4%)	3 (18%)	–
Secondary, n (%)	6 (22%)	6 (35%)	–
Superior, n (%)	20 (74%)	8 (47%)	–
MMSE (IQR)	29 (2)	27 (2.25)	$<0.01\ ^{\dagger }$
Fried score*			0.$246^{\ddagger }$
Not frail, n (%)	13 (51%)	4 (25%)	–
Pre-frail, n (%)	12 (49%)	11 (69%)	–
Frail, n (%)	0 (0%)	1 (6%)	–

Whereas, the $^{\ddagger }$ symbol represents differences computed by $\chi ^{2}$ test. BMI, body mass index; MMSE, mini-mental state examination; For each cognition group the median and the interquartile range (IQR) values are presented.

*The Fried Index is based on the Fried frailty phenotype criteria, that has been administered to 16 Controls and 16 MCIs.

## Results

### Socio-demographic data

44 older adults participated to our study. Among them, 26 were female (59%) and 18 were male (41%). Participants present an overall mean (SD) age and BMI of 70 (10) years and 25.98 (5.55) $\hbox {kg}/\hbox {m}^{2}$. Furthermore, 17 of them (39%) were diagnosed MCI. Respectively, 13 were female (76%) and 4 were male (25%). As expected, people suffering from MCI were significantly older than cognitively intact individuals ($\hbox {p}<0.01$), and have lower scores at the MMSE ($\hbox {p}<0.01$). Notwithstanding, the percentage of frail or pre-frail people did not differ between groups ($\hbox {p}=0.25$). None of them had problems understanding questions or counting numbers. All socio-demographic data and clinical information are reported in Table 1.

### Association between dual-task performance with MMSE

Results from Spearman’s correlation demonstrated that FTAP performances, both in single and dual task, and also considering the DTC, were often associated with MMSE scores. Notably, it seems that MMSE scores are more related to variation measures (standard deviation of the movement excursion and the opening velocity) and motor standard parameters (the number of tapping) at low cognitive load (CL1), while showed higher correlations to the DTC of parameters when the cognitive load increases: DTC effect on the number of tapping (CL2), on rmse of the jerk (CL2), on the SMA (CL2), on the opening velocity (CL3), on the SMA (CL3), and on the kurtosis of the acceleration (CL3). Moreover, even with less parameters, also TTHP showed some significant values. Interestingly, the MMSE score is associated with the TTHP SMA (the signal magnitude area), a measure that has been proven to be related linearly with energy expenditure (see Table 2). Notably,we attested also significant correlations between GAIT parameters and MMSE, which are reported within the supplementary material.

**Table 2:** FTAP and TTHP parameters that significantly correlates with MMSE score. The complete list of parameters extracted and evaluated is encompassed in the Supplementary Material.

Variable	Spearman’s correlation with MMSE (rho; p-value)
FTAP tap (CL1)	$\hbox {rho}=0.40$; $\hbox {p}=0.01$
FTAP excSD (CL1)	$\hbox {rho}=-0.37$; $\hbox {p}=0.01$
FTAP woSD (CL1)	$\hbox {rho}=-0.34$; $\hbox {p}=0.03$
FTAP KURT-acc (CL1)	$\hbox {rho}=-0.38$; $\hbox {p}=0.01$
FTAP KURT-acc DTC (CL1)	$\hbox {rho}=-0.33$; $\hbox {p}=0.03$
FTAP tap (CL2)	$\hbox {rho}=0.39$; $\hbox {p}=0.01$
FTAP rmse-JERK (CL2)	$\hbox {rho}=0.32$; $\hbox {p}=0.04$
FTAP KURT-acc (CL2)	$\hbox {rho}=-0.33$; $\hbox {p}=0.03$
FTAP tap DTC (CL2)	$\hbox {rho}=0.31$; $\hbox {p}=0.04$
FTAP SMA DTC (CL2)	$\hbox {rho}=-0.32$; $\hbox {p}=0.03$
FTAP rmse-JERK DTC (CL2)	$\hbox {rho}=0.32$; $\hbox {p}=0.03$
FTAP SKEW-acc (CL3)	$\hbox {rho}=0.30$; $\hbox {p}=0.04$
FTAP KURT-acc (CL3)	$\hbox {rho}=-0.36$; $\hbox {p}=0.02$
FTAP wo DTC (CL3)	$\hbox {rho}=0.33$; $\hbox {p}=0.03$
FTAP SMA DTC (CL3)	$\hbox {rho}=-0.39$; $\hbox {p}=0.01$
FTAP KURT-acc DTC (CL3)	$\hbox {rho}=-0.32$; $\hbox {p}=0.03$
TTHP SMA (CL0)	$\hbox {rho}=-0.33$; $\hbox {p}=0.03$
TTHP SMA (CL1)	$\hbox {rho}=-0.33$; $\hbox {p}=0.03$
TTHP SMA (CL2)	$\hbox {rho}=-0.41$; $\hbox {p}=0.01$
TTHP SMA (CL3)	$\hbox {rho}=-0.34$; $\hbox {p}=0.02$

### FTAP and TTHP performance in distinguishing MCI from CNA

Several FTAP, TTHP and GAIT parameters, across all the cognitive loads and also referring to the respective DTC, were able to differentiate MCI from cognitively normal subjects (see Table 3). Most notably, DT FTAP, TTHP and GAIT motor parameters, able to differentiate between cognitively unimpaired subjects and MCI, were included in several logistic regression models.

**Table 3:** FTAP, TTHP and GAIT parameters that significantly differs between CNA and MCI subjects. The complete list of parameters extracted and evaluated is encompassed in the Supplementary Material.

Variable		CNA (Mean ± SD)	MCI (Mean ± SD)	Mann–Whitney (p-value)
FTAP	excSD (CL0)	$2.11\pm 0.79$	$2.87\pm 1.28$	$<0.05$
	Taps (CL2)	$29.10\pm 16.82$	$19.77\pm 16.28$	0.04
	Correct answers (CL2)	$8.19\pm 2.62$	$5.47\pm 2.27$	$<0.01$
	woSD DTC (CL2)	$38.33\pm 65.71$	$32.17\pm 166.48$	0.02
	SKEW-acc (CL3)	$-0.93\pm 1.35$	$-1.76\pm 1.32$	0.02
	woSD DTC (CL3)	$47.90\pm 66.43$	$14.39\pm 116.36$	0.02
	Correct answers (CL3)	$5.22\pm 2.03$	$2.65\pm 1.32$	$<0.01$
TTHP	Kurt-acc (CL0)	$2.38\pm 0.91$	$2.88\pm 0.98$	0.02
	SMA (CL1)	$0.14\pm 0.01$	$0.15\pm 0.01$	0.01
	Kurt-acc (CL1)	$2.76\pm 1.11$	$3.54\pm 1.39$	0.02
	Correct answers (CL1)	$16.59\pm 3.39$	$13.35\pm 3.51$	$<0.01$
	SMA (CL2)	$0.14\pm 0.01$	$0.15\pm 0.01$	0.02
	exc DTC (CL2)	$21.39\pm 45.30$	$-5.13\pm 26.79$	$<0.05$
	Correct answers (CL2)	$8.70\pm 2.76$	$6.00\pm 2.65$	$<0.01$
	wo (CL3)	$33.48\pm 16.95$	$22.78\pm 8.40$	0.02
	SMA (CL3)	$0.14\pm 0.01$	$0.15\pm 0.01$	0.02
	exc DTC (CL3)	$24.08\pm 51.73$	$-10.14\pm 28.31$	0.01
	wo DTC (CL3)	$-6.61\pm 36.99$	$-28.17\pm 30.41$	0.04
	woSD DTC (CL3)	$34.29\pm 87.40$	$-6.61\pm 58.93$	0.02
	wc DTC (CL3)	$-22.06\pm 44.68$	$-48.80\pm 28.65$	$<0.05$
	Correct answers (CL3)	$5.33\pm 2.17$	$3.06\pm 1.14$	$<0.01$
GAIT	GSTRD (CL0)	$6.89\pm 1.12$	$7.94\pm 1.09$	$<0.01$
	GSTRD-L (CL0)	$2.24\pm 0.38$	$1.92\pm 0.26$	$<0.010$
	GSTT (CL0)	$0.07\pm 0.15$	$0.04\pm 0.02$	0.04
	GT (CL1)	$7.63\pm 1.25$	$8.29\pm 1.26$	$<0.05$
	GVEL (CL1)	$2.02\pm 0.36$	$1.85\pm 0.24$	$<0.05$
	GSRD-T DTC (CL1)	$4.10\pm 9.17$	$12.29\pm 11.24$	0.04
	GSTRD DTC (CL2)	$-48.11\pm 10.13$	$-55.23\pm 9.85$	0.01
	GSTRD-L DTC (CL2)	$256.83\pm 107.68$	$344.65\pm 117.16$	0.02
	Correct answer (CL2)	$8.07\pm 2.24$	$5.53\pm 2.76$	$<0.01$
	GSTRD (CL3)	$7.59\pm 1.47$	$8.41\pm 1.12$	0.03
	GSTRD-L (CL3)	$2.04\pm 0.36$	$1.81\pm 0.23$	0.03
	GSTT-SD DTC (CL3)	$3063.10\pm 2299.09$	$1906.18\pm 1361.25$	0.04
	Correct answer (CL3)	$5.56\pm 2.79$	$2.71\pm 1.36$	$<0.01$

**Figure 2:**
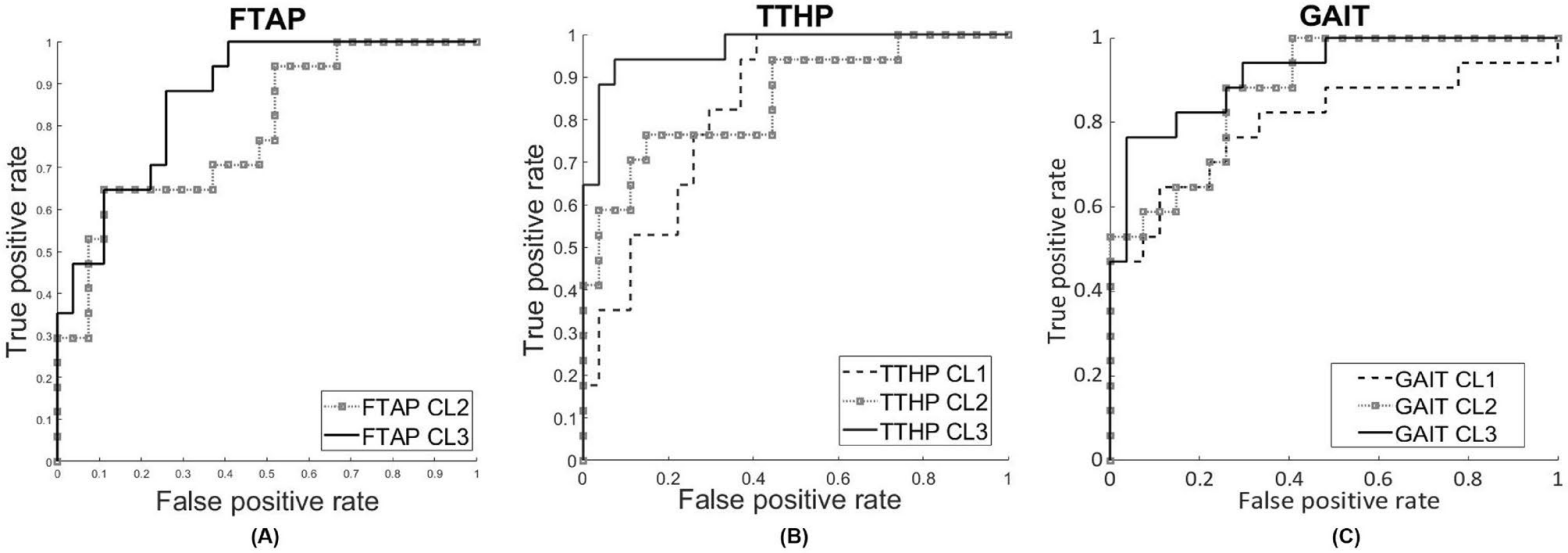
ROC curves regarding FTAP, TTHP and GAIT at CL1, CL2, and CL3.

Within our experiment, a logistic regression model on GAIT DT, also considered as the gold standard for MCDT assessment, has been able to distinguish MCI subjects from unimpaired elderly people with specificity and sensitivity of 100% and 47% ($\hbox {AUC}=0.80$) at CL1; 100% and 53% ($\hbox {AUC}=0.88$) at CL2; and 96% and 77% ($\hbox {AUC}=0.92$) at CL3. On the other hand FTAP has been able to distinguish such subjects with specificity and sensitivity of 89% and 65% at CL2 ($\hbox {AUC}=0.79$); and 89% and 65% ($\hbox {AUC}=0.87$) at CL3. No logistic regression model was built at CL1 since any FTAP parameters were not able to differentiate between subjects at such CL. Eventually, TTHP has been able to distinguish MCIs from unimpaired elderly subjects with specificity and sensitivity of 63% and 94% ($\hbox {AUC}=0.83$) at CL1; 96% and 59% ($\hbox {AUC}=0.84$) at CL2; 96% and 88% ($\hbox {AUC}=0.97$) at CL3. Importantly, age was always used as covariate in each logistic regression model (see Fig. 2).

## Discussions

### Assessing cognitive impairment using new motor and cognitive dual-task protocols

As aforementioned, we started our investigation from the comparison of FTAP and TTHP with MMSE score. Interestingly, several parameters, at each CLs, correlate in a statistically significant way with MMSE scores, using Spearman’s correlation, in order to have more reliable results in case of non-linearity in our data (see Table 2). Notwithstanding, we did not adopt any correction (i.e. age or BMI) for this computation, and this could be considered as a limit of our work. We adopt FTAP and TTHP, as well as GAIT, in order to identify MCI subjects. Several parameters were able to distinguish between such subjects. Interestingly, TTHP was able to differentiate between MCIs and unimpaired subjects at each CL, whereas FTAP did not. FTAP showed no significant differences at CL1. That could be explained by the fact that finger tapping represents a rather simple motor task, which required a more demanding cognitive task in order to highlight differences between these types of subjects (see Table 3). Eventually, statistically significant MCDT parameters were used to build logistic regression models. We aim to assess the capability of our protocols to identify MCIs. Therefore, we build 3 models for GAIT and TTHP and 2 models for FTAP, since FTAP has not been able to differentiate MCI from controls at CL1. The results, as expected, showed a great specificity for GAIT tests (ranging 100–95% along all the CLs), even though the sensitivity was not so high (47–77%). On the other hand, FTAP showed a lower specificity (89% at CL2 and CL3) and sensitivity (65% at CL2 at CL3), if compared to GAIT. Interestingly, even if the optimal operating points were equal at CL2 and CL3, regarding FTAP, the AUC at CL3 gain +8% if compared to CL2 (from 0.79 to 0.87). Conclusively, TTHP models showed a rather high specificity (ranging 63–96%), and also exhibits good sensitivity rates, (ranging 59–94%). Particularly, TTHP CL3 ROC has reveled to be the best fitting model for our purpose, with specificity and sensitivity of 96% and 88% ($\hbox {AUC}=0.97$) *vs* GAIT CL3 96% specificity and 77% sensitivity ($\hbox {AUC}=0.92$), or FTAP CL3 89% and 65% ($\hbox {AUC}=0.87$) (see Fig. 2).

### Clinical implications and future directions

In this work, we propose two novel tools for the screening of MCIs. Namely, FTAP and TTHP. These tools are based on the MCDT approach, and are proposed as alternative to the mainstream walking DT (here named GAIT). The idea behind is that: more unusual movements could engage more the subjects, but at the same time the exercises must to remain simple and possibly performed also by bedridden subjects, people with reduced mobility, or even during neuroimaging exams. Moreover, due to the wearable nature of the system, is possible to perform FTAP and TTHP basically everywhere. No additional gear (sensorized walkways^14,35^ or optoelectronic systems^19^) or ample space (hallways where is possible to walk easily) are required. Our work represents a first step in the attempt to enlarge the framework of MCDT, along with Tosizadeh et al., works^16,17^. For the best of our knowledge, there are no other works on such topic. In addition, we adopt a more refined movement analysis, taking into account fine upper and lower limbs movements. Particularly, we used information from the dominant hand finger and from the dominant foot. Nevertheless, some limitations, mainly related to sample size, are present. Further experimental trials will be scheduled to gather more data and to enlarge our sample. That could allow us to adopt more statistically robust methods. Besides, we planned to assess increasing levels of motor complexity (tapping difficulty) and their correlations to other neuropsychological tests (such as the Montreal Cognitive Assessment). In this work, we investigated the effect of an increasing cognitive load on motor performance. The same approach should be adopted also on the motor side of MCDT. That represents the strategy to highlight a sweet spot for MCI early diagnosis using novel MCDTs. Another limit related to our work concerns the extent of the clinical measure adopted. MMSE, infact, represents a screening test for global cognitive status. A more granular neurocognitive description of our subjects could be achieved, enlarging the cognitive assessment in the future works. Eventually, a certain percentage of enrolled subjects have behaved normally (psychometrically speaking) during the cognitive tests, even though report to feel a worsening of their cognitive performance. Future directions could also encompass the possibility to investigate differences between cognitively unimpaired subjects and those who report subjective cognitive decline not attested by psychometric tests, enriching the literature concerning the AD’s early diagnosis.Otherwise, it can even be applied to other clinical populations, in particular the Parkinson’s disease (in which cognitive tasks are often requested as distracting tasks to unmask the tremor)^33^, in stoke patients^36^, or even children suffering from neurodevelopmental disorders^37^. Furthermore, such protocols have also been applied to people with peripheric neuropathy^38^, and amputees^39^.

## Conclusions

In summary, within this work, we aimed at investigating the feasibility of using novel MCDT approaches to screen and assess people suffering from MCI, namely, FTAP and TTHP. The idea was to design and develop new technologies, and protocols, that would enrich the clinical repertoire with rapid, easy, and non-invasive tools. Therefore, we tested it on 44 elderly subjects, studying FTAP and TTHP performance in distinguishing between cognitively normal and MCI subjects. Moreover, we compared our new tools to gold standards for cognitive assessment and MCDT assessment, respectively MMSE and walking task (here named GAIT). Based on our previous exploration, we decided to use toe-tapping and forefinger tapping as motor counterpart for new MCDT protocols. On the other side, we decided to use counting backwards as cognitive task due to its wide usage in such field. Notwithstanding, considering the novelty of our protocols, we selected different cognitive loads (ST and DT at CL1, CL2, and CL3) to observe variations in motor performances related to an increasing cognitive load. Our findings confirm good association between FTAP and TTHP to MMSE score. Moreover, AUCs from logistic regression model we performed allow us to retain that, especially at CL3, FTAP ($\hbox {AUC}=0.87$) and TTHP ($\hbox {AUC}=0.97$) are comparable, or even better than GAIT ($\hbox {AUC}=0.92$), without needing a 10-m long hallway, and adopting simple exercises that even bedridden subjects can perform.

## Supplementary Information

Supplementary Information.
