# Supplementary material for: Innovative motor and cognitive dual-task approaches combining upper and lower limbs may improve dementia early detection
Source: Sci Rep. 2021 Apr 2;11:7449. doi: 10.1038/s41598-021-86579-3 (PMC8018979; doi:10.1038/s41598-021-86579-3)
Supplement: Supplementary file 1 — Supplementary Information. [file 41598_2021_86579_MOESM1_ESM.pdf]

Supplementary Material

Innovative Motor and Cognitive Dual-Task approaches combining upper and lower limbs may improve dementia early detection

Gianmaria Mancioppi, Laura Fiorini, Erika Rovini, Radia Zeghari, Auriane Gros,Valeria Manera, Philippe Robert, and Filippo Cavallo.

The supplementary material encompasses the full list of the neuropsychological tests adopted in this study, the clinical characterization of MCI subjects, and the data about the Motor and Cognitive Dual-Task (MCDT) protocol performed within our experiment.

1 Neuropsychological Battery and Sample Clinical Characteristic

The neuropsychological battery adopted to diagnose the subjects enclose two screening tests:

- the Mini-Mental State Examination (MMSE);
- the Frontal Assessment Battery (FAB), to avoid MMSE ceiling effect and to inspect the executive domain.

Furthermore, we assessed:

- the long-term episodic verbal memory using the Free and Cued Selective Reminding Test (FCSRT);
- the short-term verbal memory using the Digit Span (direct and inverse);
- the linguistic repertoire (and the mental flexibility) using the verbal fluency (semantic category version);

	Etiology	MCI Subtype (according to Petersen categorization)*
Subject 1	Degenerative, AD-like	amnestic single domain
Subject 2	Vascular	amnestic multiple domain
Subject 3	Degenerative, not specified	non-amnestic single domain
Subject 4	Degenerative, AD-like	amnestic multiple domain
Subject 5	Vascular	non-amnestic multiple domain
Subject 6	Degenerative, not specified	non-amnestic multiple domain
Subject 7	Missing	amnestic multiple domain
Subject 8	Degenerative, AD-like	amnestic single domain
Subject 9	Degenerative, AD-like	amnestic multiple domain
Subject 10	Vascular	amnestic multiple domain
Subject 11	Degenerative, not specified	amnestic multiple domain
Subject 12	Degenerative, not specified	non-amnestic multiple domain
Subject 13	Degenerative, AD-like	amnestic multiple domain
Subject 14	Vascular	amnestic single domain
Subject 15	Vascular	amnestic multiple domain
Subject 16	Degenerative, not specified	amnestic multiple domain
Subject 17	Missing	non-amnestic single domain

Table S 1: Subjects characterization: etiology and Petersen's categorization. *[Petersen, R. C. (2004). Mild cognitive impairment as a diagnostic entity. Journal of internal medicine, 256(3), 183-194.].

- the selective axis of attention by the Trail-Making Test (TMT) form A, and eventually the working-memory ability by the TMT form B.

Notably, every subject first underwent two screening tests (MMSE and FAB). People who performed those tests abnormally (from a psychometric point of view) were further assessed using the above-mentioned tests. We adopted the same battery with people who report a subjective cognitive decline, not attested by the screening tests. Those subjects who resulted cognitively intact at the neuropsychological exams, even complaining of subjective cognitive decline, were enclosed in the CNA group.

2 MCDT Protocol.

Concerning the MCDT protocol it provides three MCDTs, namely:

- fore-finger tapping (FTAP),
- toe-tapping heel pin (TTHP),
- walking task (GAIT).

Each MCDT have been performed both in Single (ST) and Dual-Task (DT). Notably, three different cognitive loads have been used:

- Cognitive Load 1 (CL1): performing the motor task while the subject was demanded to counting backward by 1, starting from a random number.
- Cognitive Load 2 (CL2): performing the motor task while the subject was demanded to counting backward by 3, starting from a random number.
- Cognitive Load 3 (CL3): performing the motor task while the subject was demanded to counting backward by 7, starting from a random number.

Moreover, for each CL the Cognitive Task Cost have been calculated using the following formula.

$$DTC = \frac{Parameter_{DT} - Parameter_{ST}}{Parameter_{ST}} \times 100 \qquad (1)$$

Parameters. Hereinafter (Table S 2), are reported parameters' names, their definitions, the means and standard deviations (SD) related to extracted parameters for Cognitive Normal Adult (CNA) and Mild Cognitive Impairment (MCI) subjects, P-value related to Mann-Whitney test and Spearman's Correlation are also reported, and eventually, the Spearman's Correlation rho is reported as well.

Table S 2: Summary Table.

Parameters	Definition	CNA (mean, SD)	MCI (mean SD)	Mann-Whitney (p-value)	Spearman Correlation with MMSE (rho, p-value)
**FTAP**					
**CL0**					
Tap	Number of Tapping	43.07±12.29	40.77±12.24	p=0.7265	rho=0.20; p=0.19
exc	Excursion	13.08± 6.57	14.54±6.96	p=0.4921	rho=-0.25; p=0.10
excSD	Excursion SD	2.11± 0.79	2.87±1.28	p=0.0467	rho=-0.13; p=0.39
wo	Opening Velocity	67.90±35.59	74.28±28.80	p=0.2889	rho=-0.17; p=0.27
woSD	Opening Velocity SD	10.37±4.94	15.42±8.91	p=0.1289	rho=-0.05; p=0.73
wc	Closing Velocity	69.57±36.18	71.35±36.95	p=0.6998	rho=-0.06; p=0.70
wcSD	Closing Velocity SD	15.41±5.65	20.74±13.02	p=0.3114	rho=-0.06; p=0.70
SMA	Signal Magnitude Area	0.12±0.01	0.12±0.01	p=0.5679	rho=0.09; p=0.55
rmse-JERK	Jerk Root Mean Squared Error	53.22±27.89	56.40±23.65	p=0.4056	rho=0.02; p=0.89
SKEW-acc	Acceleration Skewness	-0.16±0.34	-0.12±0.23	p=0.9231	rho=-0.15; p=0.33
KURT-acc	Acceleration Kurtosis	2.24±0.69	2.36± 1.39	p=0.8565	rho=0.09; p=0.55
**CL1**					
Tap	Number of Tapping	34.15±15.37	27.47±13.93	p=0.1254	rho=0.40; p<0.01
exc	Excursion	13.65±6.63	15.67±6.76	p=0.3855	rho=-0.28; p=0.07
excSD	Excursion SD	2.40±1.09	2.91±1.32	p=0.1481	rho=-0.37; p=0.01
wo	Opening Velocity	58.90±32.37	59.06±20.11	p=0.4549	rho=-0.12; p=0.43
woSD	Opening Velocity SD	11.96± 6.19	14.51±7.94	p=0.2889	rho=-0.34; p=0.03
wc	Closing Velocity	54.84±36.50	45.77±21.04	p=0.6470	rho=0.11; p=0.49
wcSD	Closing Velocity SD	16.54±7.20	18.59±8.39	p=0.5468	rho=-0.16; p=0.31
SMA	Signal Magnitude Area	0.12±0.01	0.12±0.01	p=0.8956	rho=-0.07; p=0.64

Table S 2: Summary Table.

Parameters	Definition	CNA (mean, SD)	MCI (mean SD)	Mann-Whitney (p-value)	Spearman Correlation with MMSE (rho, p-value)
rmse-JERK	Jerk Root Mean Squared Error	42.71±25.01	40.23±18.46	p=0.9424	rho=0.19; p=0.21
SKEW-acc	Acceleration Skewness	-0.37±0.55	-0.79±0.71	p=0.0652	rho=0.23; p=0.14
KURT-acc	Acceleration Kurtosis	3.06±1.44	4.69±2.99	p=0.0670	rho=-0.38; p=0.01
Tap-DTC	Number of Tapping DTC	-19.52±27.66	-29.57±32.45	p=0.1414	rho=0.28; p=0.06
exc-DTC	Excursion DTC	12.34±62.39	12.81±31.29	p=0.4696	rho=-0.09; p=0.58
excSD-DTC	Excursion SD DTC	22.67±66.01	18.67±67.00	p=0.7724	rho=-0.16; p=0.31
wo-DTC	Opening Velocity DTC	-11.40±18.88	-14.37±29.09	p=0.8095	rho=0.17; p=0.26
woSD-DTC	Opening Velocity SD DTC	19.17±50.09	28.70±148.32	p=0.1415	rho=-0.14; p=0.37
wc-DTC	Closing Velocity DTC	-23.16±26.84	-13.25±66.31	p=0.7177	rho=0.19; p=0.22
wcSD-DTC	Closing Velocity SD DTC	10.11±42.79	156.21±639.81	p=0.6820	rho=-0.08; p=0.61
SMA-DTC	Signal Magnitude Area DTC	0.52±5.97	2.2302±5.76	p=0.2286	rho=-0.22; p=0.14
rmse-JERK-DTC	Jerk Root Mean Squared Error DTC	-15.39±30.82	-20.64±37.71	p=0.3855	rho=0.26; p=0.09
SKEW-acc-DTC	Acceleration Skewness DTC	-0.10±731.10	-Inf±NaN	p=0.7724	rho=0.02; p=0.91
KURT-acc-DTC	Acceleration Kurtosis DTC	41.54±72.85	124.96±168.91	p=0.0538	rho=-0.33; p=0.03
**CL2**					
Tap	Number of Tapping	29.41±16.82	19.77±16.28	p=0.0358	rho=0.39; p<0.01
exc	Excursion	12.88±5.23	14.34±6.56	p=0.6556	rho=-0.17; p=0.27
excSD	Excursion SD	2.85±1.55	2.99±2.10	p=0.9328	rho=-0.14; p=0.36
wo	Opening Velocity	55.09±29.46	50.89±23.36	p=0.7358	rho=0.06; p=0.70
woSD	Opening Velocity SD	13.46±7.15	14.18±7.91	p=0.6644	rho=-0.21; p=0.18
wc	Closing Velocity	46.88±35.13	32.29±24.68	p=0.2101	rho=0.24; p=0.12
wcSD	Closing Velocity SD	17.44±9.94	16.31±11.25	p=0.7358	rho=-0.06; p=0.70

Table S 2: Summary Table.

	Parameters	Definition	CNA (mean, SD)	MCI (mean SD)	Mann-Whitney (p-value)	Spearman Correlation with MMSE (rho, p-value)
	SMA	Signal Magnitude Area	0.12±0.01	0.12±0.01	p=0.9496	rho=-0.11; p=0.48
	rmse-JERK	Jerk Root Mean Squared Error	40.09±27.58	33.26±21.56	p=0.4125	rho=0.32; p=0.04
	SKEW-acc	Acceleration Skewness	-0.83±1.13	-1.36±1.31	p=0.1258	rho=0.16; p=0.29
	KURT-acc	Acceleration Kurtosis	5.76±5.88	8.46±6.93	p=0.0870	rho=-0.33; p=0.03
	Tap-DTC	Number of Tapping DTC	-0.03±0.03	-0.05±0.05	p=0.0939	rho=0.31; p=0.04
	exc-DTC	Excursion DTC	10.74±57.20	4.24±35.95	p=0.7724	rho=0.12; p=0.43
	excSD-DTC	Excursion SD DTC	49.53±104.65	22.98±132.90	p=0.1063	rho=0.11; p=0.50
	wo-DTC	Opening Velocity DTC	-15.55±22.52	-26.96±30.14	p=0.2473	rho=0.28; p=0.06
	woSD-DTC	Opening Velocity SD DTC	38.33± 65.71	32.17±166.48	p=0.0182	rho=0.11; p=0.47
	wc-DTC	Closing Velocity DTC	-34.06±33.08	-32.69±88.87	p=0.1172	rho=0.33; p=0.03
	wcSD-DTC	Closing Velocity SD DTC	17.49±59.66	131.87±625.19	p=0.1771	rho=0.11; p=0.48
	SMA-DTC	Signal Magnitude Area DTC	>0.01±0.01	>0.01±0.01	p=0.3982	rho=-0.32; p=0.03
	rmse-JERK-DTC	Jerk Root Mean Squared Error DTC	-0.02±0.04	-0.03±0.05	p=0.1351	rho=0.34; p=0.02
	SKEW-acc-DTC	Acceleration Skewness DTC	-0.11±1.12	-Inf±NaN	p=0.4056	rho=0.09; p=0.54
	KURT-acc-DTC	Acceleration Kurtosis DTC	0.17±0.27	0.31±0.33	p=0.1229	rho=-0.26; p=0.09
**CL3**						
	Tap	Number of Tapping	27.19±18.55	19.35±	p=0.1886	rho=0.28; p=0.07
	exc	Excursion	13.54±5.75	13.67±6.28	p=0.8755	rho=-0.08; p=0.63
	excSD	Excursion SD	2.88±1.90	2.76±1.47	p=1.0000	rho=-0.15; p=0.34
	wo	Opening Velocity	53.82±27.79	44.61±18.55	p=0.3597	rho=0.15; p=0.33
	woSD	Opening Velocity SD	13.52±7.26	13.18±6.62	p=0.8471	rho=-0.05; p=0.73
	wc	Closing Velocity	44.70±36.44	28.77±23.61	p=0.1849	rho=0.22; p=0.16

Table S 2: Summary Table.

Parameters	Definition	CNA (mean, SD)	MCI (mean SD)	Mann-Whitney (p-value)	Spearman Correlation with MMSE (rho, p-value)
wcSD	Closing Velocity SD	16.62±11.77	17.03±11.88	p=0.8660	rho=-0.13; p=0.41
SMA	Signal Magnitude Area	0.12±0.01	0.12±0.01	p=0.7591	rho=-0.19; p=0.21
rmse-JERK	Jerk Root Mean Squared Error	38.22±25.97	32.99±18.63	p=0.6998	rho=0.32; p=0.03
SKEW-acc	Acceleration Skewness	-0.93±1.35	-1.76±1.32	p=0.0154	rho=0.30; p=0.04
KURT-acc	Acceleration Kurtosis	6.88± 7.37	10.23±8.14	p=0.1089	rho=-0.36; p=0.02
Tap-DTC	Number of Tapping DTC	-0.04±0.04	-0.05±0.04	p=0.2328	rho=0.19; p=0.21
exc-DTC	Excursion DTC	15.61±56.16	-1.67±34.95	p=0.3350	rho=0.17; p=0.27
excSD-DTC	Excursion SD DTC	48.58±91.30	6.64±57.35	p=0.0916	rho=-0.03; p=0.87
wo-DTC	Opening Velocity DTC	-15.39±30.89	-33.53±32.01	p=0.0635	rho=0.33; p=0.03
woSD-DTC	Opening Velocity SD DTC	41.90±66.43	14.39±116.36	p=0.0235	rho=0.07; p=0.66
wc-DTC	Closing Velocity DTC	-37.01±42.05	-35.07±84.62	p=0.3114	rho=0.21; p=0.16
wcSD-DTC	Closing Velocity SD DTC	13.54±77.02	127.51±585.86	p=0.4264	rho=-0.08; p=0.59
SMA-DTC	Signal Magnitude Area DTC	>0.01±0.01	>0.01±0.01	p=0.7416	rho=-0.39; p=0.01
rmse-JERK-DTC	Jerk Root Mean Squared Error DTC	-0.2±0.04	-0.03±0.04	p=0.4696	rho=0.25; p=0.10
SKEW-acc-DTC	Acceleration Skewness DTC	-0.16±1.47	-inf±NaN	p=0.9808	rho=-0.06; p=0.68
KURT-acc-DTC	Acceleration Kurtosis DTC	0.22±0.33	0.40±0.42	p=0.1415	rho=-0.32; p=0.03
**TTHP**					
**CL0**					
Tap	Number of Tapping	38.26±9.29	33.88±7.13	p=0.1438	rho=0.26; p=0.09
exc	Excursion	8.40±3.81	8.43±3.57	p=0.9808	rho=-0.09; p=0.56
excSD	Excursion SD	1.01±0.42	1.38±0.97	p=0.3289	rho=-0.03; p=0.83
wo	Opening Velocity	38.90±19.56	34.33±13.03	p=0.6470	rho=0.02; p=0.89

Table S 2: Summary Table.

Parameters	Definition	CNA (mean, SD)	MCI (mean SD)	Mann-Whitney (p-value)	Spearman Correlation with MMSE (rho, p-value)
woSD	Opening Velocity SD	4.93±2.80	6.19±3.88	p=0.2675	rho=-0.03; p=0.83
wc	Closing Velocity	43.23±21.35	38.81±17.67	p=0.5629	rho=0.04; p=0.81
wcSD	Closing Velocity SD	5.85±2.95	7.92±5.48	p=0.1732	rho=-0.02; p=0.90
SMA	Signal Magnitude Area	0.15±0.01	0.15±0.01	p=0.0582	rho=-0.33; p=0.03
rmse-JERK	Jerk Root Mean Squared Error	36.24± 21.36	29.71±11.48	p=0.5230	rho=0.14; p=0.38
SKEW-acc	Acceleration Skewness	0.37±0.31	0.39±0.50	p=0.9712	rho=0.05; p=0.74
KURT-acc	Acceleration Kurtosis	2.38±0.91	2.88±0.98	p=0.0213	rho=-0.20; p=0.20
**CL1**					
Tap	Number of Tapping	31.89±12.01	27.47±7.76	p=0.3524	rho=0.12; p=0.45
exc	Excursion	9.53±4.35	8.40±3.64	p=0.2889	rho=-0.11; p=0.47
excSD	Excursion SD	1.08±0.51	1.32±0.61	p=0.2779	rho=-0.06; p=0.69
wo	Opening Velocity	36.26±17.04	29.57±9.38	p=0.2281	rho=-0.01; p=0.95
woSD	Opening Velocity SD	5.15±3.10	5.61±2.72	p=0.5308	rho=-0.04; p=0.77
wc	Closing Velocity	41.69±21.35	30.91±14.96	p=0.1144	rho=0.05; p=0.72
wcSD	Closing Velocity SD	8.61±5.49	8.31±4.66	p=0.8945	rho=0.05; p=0.73
SMA	Signal Magnitude Area	0.14±0.01	0.15±0.01	p=0.0117	rho=-0.33; p=0.03
rmse-JERK	Jerk Root Mean Squared Error	31.64±18.87	24.10±9.02	p=0.4334	rho=0.03; p=0.85
SKEW-acc	Acceleration Skewness	0.33±0.40	0.35±0.69	p=0.8755	rho=-0.11; p=0.48
KURT-acc	Acceleration Kurtosis	2.76±1.11	3.55±1.39	p=0.0234	rho=-0.21; p=0.16
Tap-DTC	Number of Tapping DTC	-15.92±23.60	-17.89±19.54	p=0.6819	rho=0.02; p=0.92
exc-DTC	Excursion DTC	16.45±34.85	2.36±19.97	p=0.2473	rho=-0.11; p=0.47
excSD-DTC	Excursion SD DTC	14.27±58.17	23.20±62.27	p=0.5309	rho=-0.13; p=0.39

Table S 2: Summary Table.

Parameters	Definition	CNA (mean, SD)	MCI (mean SD)	Mann-Whitney (p-value)	Spearman Correlation with MMSE (rho, p-value)
wo-DTC	Opening Velocity DTC	-2.11±32.74	-9.72±22.91	p=0.3725	rho=-0.08; p=0.60
woSD-DTC	Opening Velocity SD DTC	13.99±66.34	3.77±41.18	p=0.9232	rho=-0.07; p=0.66
wc-DTC	Closing Velocity DTC	-1.52±29.82	-16.98±22.15	p=0.0827	rho=0.03; p=0.83
wcSD-DTC	Closing Velocity SD DTC	61.84±92.21	22.29±64.43	p=0.1621	rho=0.05; p=0.75
SMA-DTC	Signal Magnitude Area DTC	-1.47±2.82	-0.74±2.09	p=0.3605	rho=-0.03; p=0.84
rmse-JERK-DTC	Jerk Root Mean Squared Error DTC	-8.23±30.68	-13.50±32.37	p=0.3597	rho=0.01; p=0.94
SKEW-acc-DTC	Acceleration Skewness DTC	-9.63±138.40	-90.64±320.50	p=0.7087	rho=0.00; p=0.98
KURT-acc-DTC	Acceleration Kurtosis DTC	18.01±31.49	26.64±39.19	p=0.5468	rho=-0.04; p=0.78
**CL2**					
Tap	Number of Tapping	27.19±14.58	22.53±11.40	p=0.3226	rho=0.15; p=0.32
exc	Excursion	9.81±4.74	7.95±4.06	p=0.2014	rho=0.08; p=0.61
excSD	Excursion SD	1.47±1.21	1.41±0.72	p=0.8660	rho=-0.06; p=0.71
wo	Opening Velocity	33.54±15.51	24.65±7.84	p=0.0725	rho=0.15; p=0.34
woSD	Opening Velocity SD	5.33±3.12	5.57±2.59	p=0.6820	rho=-0.15; p=0.34
wc	Closing Velocity	32.74±20.02	20.54±10.47	p=0.0635	rho=0.15; p=0.32
wcSD	Closing Velocity SD	9.64±6.06	7.78±5.66	p=0.2573	rho=0.09; p=0.55
SMA	Signal Magnitude Area	0.14±0.01	0.15±0.01	p=0.0182	rho=-0.41; p=0.01
rmse-JERK	Jerk Root Mean Squared Error	28.55±17.67	19.53±9.10	p=0.1012	rho=0.20; p=0.19
SKEW-acc	Acceleration Skewness	0.35±0.55	0.60±0.67	p=0.3171	rho=-0.06; p=0.72
KURT-acc	Acceleration Kurtosis	4.05±2.73	5.61±3.44	p=0.0785	rho=-0.19; p=0.21
Tap-DTC	Number of Tapping DTC	-28.75±31.38	-32.90±30.69	p=0.6819	rho=0.11; p=0.46
exc-DTC	Excursion DTC	21.39±45.30	-5.13±26.79	p=0.0454	rho=0.24; p=0.12

Table S 2: Summary Table.

Parameters	Definition	CNA (mean, SD)	MCI (mean SD)	Mann-Whitney (p-value)	Spearman Correlation with MMSE (rho, p-value)
excSD-DTC	Excursion SD DTC	54.73±117.51	25.96±64.77	p=0.6298	rho=-0.15; p=0.33
wo-DTC	Opening Velocity DTC	-7.31±32.09	-22.76±27.16	p=0.1481	rho=0.19; p=0.21
woSD-DTC	Opening Velocity SD DTC	17.03±59.41	5.65±42.08	p=0.8660	rho=-0.25; p=0.11
wc-DTC	Closing Velocity DTC	-19.13±40.17	-41.89±26.36	p=0.1012	rho=0.24; p=0.12
wcSD-DTC	Closing Velocity SD DTC	88.41±118.77	19.86±78.40	p=0.0670	rho=0.01; p=0.94
SMA-DTC	Signal Magnitude Area DTC	-0.96±3.08	-0.34±2.76	p=0.5047	rho=-0.16; p=0.31
rmse-JERK-DTC	Jerk Root Mean Squared Error DTC	-15.63±32.96	-27.22±38.92	p=0.2281	rho=0.16; p=0.30
SKEW-acc-DTC	Acceleration Skewness DTC	-22.41±277.44	7.76±297.25	p=0.2190	rho=-0.04; p=0.82
KURT-acc-DTC	Acceleration Kurtosis DTC	78.55±123.42	97.32±99.69	p=0.5468	rho=-0.07; p=0.64
**CL3**					
Tap	Number of Tapping	26.63±16.64	21.88±15.24	p=0.3785	rho=0.17; p=0.26
exc	Excursion	9.96±5.06	7.57±4.11	p=0.1116	rho=0.12; p=0.42
excSD	Excursion SD	1.51±0.99	1.27±0.67	p=0.4997	rho=-0.09; p=0.57
wo	Opening Velocity	33.48±16.95	22.78±8.40	p=0.0194	rho=0.17; p=0.28
woSD	Opening Velocity SD	5.71±3.16	4.76±1.91	p=0.4696	rho=-0.10; p=0.53
wc	Closing Velocity	31.74±23.07	17.75±11.08	p=0.0706	rho=0.20; p=0.20
wcSD	Closing Velocity SD	8.47±5.21	7.35±5.12	p=0.3350	rho=0.01; p=0.94
SMA	Signal Magnitude Area	0.14±0.01	0.15±0.01	p=0.0185	rho=-0.34; p=0.02
rmse-JERK	Jerk Root Mean Squared Error	28.11±19.13	18.33±10.14	p=0.1289	rho=0.16; p=0.32
SKEW-acc	Acceleration Skewness	0.31±0.80	0.70±1.06	p=0.5308	rho=0.01; p=0.96
KURT-acc	Acceleration Kurtosis	5.00±4.21	9.22±10.54	p=0.111	rho=-0.19; p=0.21
Tap-DTC	Number of Tapping DTC	-30.93±36.23	-35.96±40.00	p=0.5959	rho=0.13; p=0.40

Table S 2: Summary Table.

Parameters	Definition	CNA (mean, SD)	MCI (mean SD)	Mann-Whitney (p-value)	Spearman Correlation with MMSE (rho, p-value)
exc-DTC	Excursion DTC	24.08±51.73	-10.14±28.31	p=0.0149	rho=0.28; p=0.06
excSD-DTC	Excursion SD DTC	59.04±106.39	25.74±88.47	p=0.1931	rho=-0.20; p=0.20
wo-DTC	Opening Velocity DTC	-6.61±36.99	-28.17±30.41	p=0.0429	rho=0.21; p=0.18
woSD-DTC	Opening Velocity SD DTC	34.29±87.40	-6.61±58.93	p=0.0170	rho=-0.02; p=0.88
wc-DTC	Closing Velocity DTC	-22.06±44.68	-48.80±28.65	p=0.0454	rho=0.30; p=0.05
wcSD-DTC	Closing Velocity SD DTC	78.34±140.71	3.68±71.97	p=0.1063	rho=-0.12; p=0.45
SMA-DTC	Signal Magnitude Area DTC	-0.92±3.00	-0.74±2.09	p=0.7605	rho=0.04; p=0.78
rmse-JERK-DTC	Jerk Root Mean Squared Error DTC	-17.48±37.15	-31.59±43.96	p=0.1012	rho=0.17; p=0.26
SKEW-acc-DTC	Acceleration Skewness DTC	-46.99±442.67	-47.27±430.70	p=0.6731	rho=0.04; p=0.79
KURT-acc-DTC	Acceleration Kurtosis DTC	126.05±201.26	219.26±328.49	p=0.6470	rho=-0.09; p=0.56
**GAIT**					
**CL0**					
GT	Gait Time	8.79±1.71	9.94±2.04	p=0.08	rho=-0.31;p=0.04
GSTRD	Gait Stride	6.89±1.12	7.94±1.09	p<0.01	rho=-0.34; p=0.02
GVEL	Gait Velocity	1.77±0.33	1.57±0.29	p=0.09	rho=0.30; p=0.05
GSTRD-L	Gait Stride Length	2.24±0.38	1.92±0.26	p<0.01	rho=0.34; p=0.02
GSTRD-H	Gait Stride Height	0.10±0.04	0.08±0.02	p=0.32	rho=0.17; p=0.26
GSTRD-H-SD	Gait Stride Height SD	0.04±0.04	0.03±0.01	p=0.83	rho=0.06; p=0.69
GSTRD-T	Gait Stride Time	1.15±0.18	1.14±0.14	p=0.89	rho=-0.12; p=0.46
GSTRD-T-SD	Gait Stride Time SD	0.09±0.15	0.04±0.06	p=0.73	rho=-0.33; p=0.03
GSWT	Gait Swing Time	0.51±0.05	0.50±0.07	p=0.59	rho=-0.08; p=0.59
GSWT-SD	Gait Swing Time SD	0.04±0.06	0.02±0.02	p=0.98	rho=-0.08; p=0.63

Table S 2: Summary Table.

Parameters	Definition	CNA (mean, SD)	MCI (mean SD)	Mann-Whitney (p-value)	Spearman Correlation with MMSE (rho, p-value)
GSTT	Gait Stance Time	0.64±0.17	0.64±0.10	p=0.39	rho=-0.12; p=0.45
GSTT-SD	Gait Stance Time SD	0.07±0.15	0.04±0.02	p=0.04	rho=-0.24; p=0.12
GRS	Gait Relative Stance	54.90±4.91	56.29±4.43	p=0.10	rho=-0.14;p=0.35
GEXC	Gait Excursion	83.78±10.22	83.02±9.97	p=0.97	rho=0.05; p=0.76
GEXC-SD	Gait Excursion SD	3.63±1.71	3.32±2.36	p=0.29	rho=0.27; p=0.08
GLAT	Gait Latency	3.57±0.48	3.46±0.56	p=0.59	rho=-0.09; p=0.55
**CL1**					
GT	Gait Time	7.63±1.25	8.29±1.26	p<0.05	rho=-0.30; p=0.05
GSTRD	Gait Stride	1.57±0.35	1.36±0.31	p=0.21	rho=-0.22; p=0.14
GVEL	Gait Velocity	2.02±0.36	1.85±0.24	p<0.05	rho=0.30; p=0.05
GSTRD-L	Gait Stride Length	0.09±0.03	0.09±0.04	p=0.21	rho=0.22; p=0.14
GSTRD-H	Gait Stride Height	0.03±0.02	0.02±0.01	p=0.63	rho=-0.06; p=0.69
GSTRD-H-SD	Gait Stride Height SD	1.19±0.18	1.28±0.22	p=0.91	rho=-0.02; p=0.91
GSTRD-T	Gait Stride Time	0.05±0.03	0.11±0.14	p=0.17	rho=-0.21; p=0.17
GSTRD-T-SD	Gait Stride Time SD	0.53±0.06	0.55±0.11	p=0.11	rho=-0.19; p=0.22
GSWT	Gait Swing Time	0.03±0.02	0.04±0.03	p=0.36	rho=-0.25; p=0.11
GSWT-SD	Gait Swing Time SD	0.66±0.13	0.73±0.16	p=0.17	rho=-0.12; p=0.42
GSTT	Gait Stance Time	0.03±0.03	0.09±0.17	p=0.10	rho=-0.17; p=0.27
GSTT-SD	Gait Stance Time SD	54.97±2.82	57.18±5.87	p=0.11	rho=-0.11; p=0.47
GRS	Gait Relative Stance	80.94±12.79	79.07±9.99	p=0.39	rho=-0.02; p=0.89
GEXC	Gait Excursion	3.38±1.91	3.40±1.53	p=0.81	rho=0.07; p=0.63
GEXC-SD	Gait Excursion SD	3.50±0.49	3.50±0.61	p=0.62	rho=0.08; p=0.63

Table S 2: Summary Table.

	Parameters	Definition	CNA (mean, SD)	MCI (mean SD)	Mann-Whitney (p-value)	Spearman Correlation with MMSE (rho, p-value)
	GLAT	Gait Latency	12.34±5.23	14.30±8.96	p=0.82	rho=-0.18; p=0.23
	GT-DTC	Gait Time DTC	14.45±14.33	17.38±16.86	p=0.43	rho=-0.10; p=0.50
	GSTRD-DTC	Gait Stride DTC	12.52±22.48	5.06±12.79	p=0.39	rho=0.11; p=0.47
	GVEL-DTC	Gait Velocity DTC	-11.29±10.69	-13.03±13.58	p=0.41	rho=0.10; p=0.51
	GSTRD-L-DTC	Gait Stride Length DTC	-8.32±14.65	-3.45±11.578	p=0.39	rho=-0.11; p=0.47
	GSTRD-H-DTC	Gait Stride Height DTC	0.62±32.52	11.53±35.91	p=0.37	rho=-0.25; p=0.10
	GSTRD-H-SD-DTC	Gait Stride Height SD DTC	12.88±85.86	1.57±57.50	p=0.68	rho=-0.04; p=0.81
	GSTRD-T-DTC	Gait Stride Time DTC	4.10±9.71	12.29±11.24	p=0.04	rho=-0.18; p=0.24
	GSTRD-T-SD-DTC	Gait Stride Time SD DTC	73.63±214.05	220.67±464.72	p=0.16	rho=0.03; p=0.85
	GSWT-DTC	Gait Swing Time DTC	4.50±9.91	9.63±10.00	p=0.15	rho=-0.27; p=0.08
	GSWT-SD-DTC	Gait Swing Time SD DTC	Inf±NaN	98.66±151.96	p=0.20	rho=0.00; p=0.99
	GSTT-DTC	Gait Stance Time DTC	4.72±11.90	14.10±14.26	p=0.08	rho=-0.14; p=0.37
	GSTT-SD-DTC	Gait Stance Time SD DTC	Inf±NaN	145.20±387.48	p=0.43	rho=0.06; p=0.70
	GRS-DTC	Gait Relative Stance DTC	0.51±5.94	1.47±3.64	p=0.94	rho=0.17; p=0.27
	GEXC-DTC	Gait Excursion DTC	-3.69±5.67	-4.57±6.74	p=0.81	rho=0.02; p=0.90
	GEXC-SD-DTC	Gait Excursion SD DTC	7.96±74.36	63.39±184.16	p=0.27	rho=-0.17; p=0.28
	GLAT-DTC	Gait Latency DTC	-1.11±13.82	3.93±27.51	p=0.90	rho=-0.02; p=0.88
**CL2**						
	GT	Gait Time	12.34±5.23	14.30±8.96	p=0.43	rho=-0.33; p=0.03
	GSTRD	Gait Stride	7.67±1.47	8.29±1.05	p=0.11	rho=-0.31; p=0.04
	GVEL	Gait Velocity	1.38±0.43	1.25±0.38	p=0.43	rho=0.33; p=0.03
	GSTRD-L	Gait Stride Length	2.03±0.39	1.84±0.21	p=0.11	rho=0.31; p=0.04

Table S 2: Summary Table.

**Parameters**	**Definition**	**CNA (mean, SD)**	**MCI (mean SD)**	**Mann-Whitney (p-value)**	**Spearman Correlation with MMSE (rho, p-value)**
GSTRD-H	Gait Stride Height	0.11±0.06	0.10±0.05	p=0.67	rho=0.05; p=0.77
GSTRD-H-SD	Gait Stride Height SD	0.04±0.05	0.04±0.03	p=0.20	rho=-0.15; p=0.32
GSTRD-T	Gait Stride Time	1.48±0.53	1.57±0.77	p=0.76	rho=-0.21; p=0.16
GSTRD-T-SD	Gait Stride Time SD	0.20±0.28	0.25±0.50	p=0.70	rho=-0.23; p=0.13
GSWT	Gait Swing Time	0.62±0.17	0.61±0.17	p=0.91	rho=-0.19; p=0.21
GSWT-SD	Gait Swing Time SD	0.10±0.17	0.06±0.07	p=0.38	rho=-0.15; p=0.32
GSTT	Gait Stance Time	0.86±0.38	0.96±0.63	p=0.46	rho=-0.15; p=0.34
GSTT-SD	Gait Stance Time SD	0.15±0.24	0.22±0.49	p=0.57	rho=-0.16; p=0.29
GRS	Gait Relative Stance	56.87±4.51	59.14±6.61	p=0.28	rho=-0.07; p=0.67
GEXC	Gait Excursion	79.90±12.67	78.97±11.58	p=0.94	rho=0.12; p=0.43
GEXC-SD	Gait Excursion SD	4.49±4.20	4.90±2.22	p=0.06	rho=-0.29; p=0.06
GLAT	Gait Latency	4.16±1.00	3.75±0.75	p=0.11	rho=-0.24; p=0.12
GT-DTC	Gait Time DTC	-61.87±19.89	-64.51±16.25	p=0.68	rho=0.18; p=0.25
GSTRD-DTC	Gait Stride DTC	-48.11±10.13	-55.23±9.85	p=0.01	rho=0.16; p=0.31
GVEL-DTC	Gait Velocity DTC	671.23±502.43	921.75±881.53	p=0.24	rho=-0.35; p=0.02
GSTRD-L-DTC	Gait Stride Length DTC	256.83±107.68	344.65±117.16	p=0.02	rho=-0.33; p=0.03
GSTRD-H-DTC	Gait Stride Height DTC	1579.03±671.73	1566.93±749.32	p=0.96	rho=0.09; p=0.55
GSTRD-H-SD-DTC	Gait Stride Height SD DTC	9413.51±6509.43	8162.79±2820.60	p=0.70	rho=0.05; p=0.74
GSTRD-T-DTC	Gait Stride Time DTC	-90.88±4.65	-91.29±3.89	p=0.68	rho=0.07; p=0.63
GSTRD-T-SD-DTC	Gait Stride Time SD DTC	0.64±89.70	27.28±115.98	p=0.43	rho=0.09; p=0.55
GSWT-DTC	Gait Swing Time DTC	189.07±93.90	217.73±152.55	p=0.40	rho=-0.15; p=0.33
GSWT-SD-DTC	Gait Swing Time SD DTC	Inf±NaN	1166.30±2487.98	p=0.65	rho=-0.15; p=0.32

Table S 2: Summary Table.

	Parameters	Definition	CNA (mean, SD)	MCI (mean SD)	Mann-Whitney (p-value)	Spearman Correlation with MMSE (rho, p-value)
	GSTT-DTC	Gait Stance Time DTC	-0.22±24.30	-4.79±21.64	p=0.80	rho=-0.08; p=0.62
	GSTT-SD-DTC	Gait Stance Time SD DTC	Inf±NaN	108.73±262.77	p=0.11	rho=0.03; p=0.83
	GRS-DTC	Gait Relative Stance DTC	-98.46±0.59	-98.31±1.04	p=0.96	rho=-0.11; p=0.49
	GEXC-DTC	Gait Excursion DTC	-99.82±0.30	-99.72±0.62	p=0.55	rho=-0.17; p=0.27
	GEXC-SD-DTC	Gait Excursion SD DTC	1853.15±1013.67	3046.01±3372.69	p=0.18	rho=-0.23; p=0.13
	GLAT-DTC	Gait Latency DTC	2189.52±550.91	2231.27±467.24	p=0.66	rho=0.14; p=0.36
**CL3**						
	GT	Gait Time	13.41±7.28	12.91±3.02	p=0.32	rho=-0.26; p=0.08
	GSTRD	Gait Stride	7.59±1.47	8.41±1.12	p=0.03	rho=-0.29; p=0.06
	GVEL	Gait Velocity	1.34±0.47	1.22±0.26	p=0.31	rho=0.27; p=0.08
	GSTRD-L	Gait Stride Length	2.04±0.36	1.81±0.23	p=0.03	rho=0.29; p=0.06
	GSTRD-H	Gait Stride Height	0.11±0.05	0.09±0.05	p=0.23	rho=0.16; p=0.30
	GSTRD-H-SD	Gait Stride Height SD	0.05±0.04	0.04±0.03	p=0.36	rho=0.13; p=0.42
	GSTRD-T	Gait Stride Time	1.61±0.75	1.42±0.30	p=1	rho=-0.16; p=0.29
	GSTRD-T-SD	Gait Stride Time SD	0.26±0.44	0.11±0.14	p=0.31	rho=-0.11; p=0.48
	GSWT	Gait Swing Time	0.64±0.20	0.61±0.16	p=0.86	rho=-0.19; p=0.21
	GSWT-SD	Gait Swing Time SD	0.08±0.09	0.08±0.15	p=0.44	rho=-0.12; p=0.43
	GSTT	Gait Stance Time	0.97±0.58	0.82±0.15	p=0.66	rho=-0.15; p=0.34
	GSTT-SD	Gait Stance Time SD	0.26±0.46	0.06±0.04	p=0.48	rho=-0.18; p=0.26
	GRS	Gait Relative Stance	58.23±6.35	57.74±4.12	p=0.58	rho=-0.05; p=0.74
	GEXC	Gait Excursion	79.20±13.16	77.46±12.02	p=0.60	rho=0.04; p=0.78
	GEXC-SD	Gait Excursion SD	4.80±4.27	4.22±1.85	p=0.50	rho=0.06; p=0.70

Table S 2: Summary Table.

**Parameters**	**Definition**	**CNA (mean, SD)**	**MCI (mean SD)**	**Mann-Whitney (p-value)**	**Spearman Correlation with MMSE (rho, p-value)**
GLAT	Gait Latency	3.98±1.27	3.66±0.54	p=0.32	rho=-0.18; p=0.23
GT-DTC	Gait Time DTC	849.44±268.17	726.56±204.14	p=0.33	rho=0.26; p=0.09
GSTRD-DTC	Gait Stride DTC	-34.28±65.46	-37.66±28.66	p=0.25	rho=-0.21; p= 0.17
GVEL-DTC	Gait Velocity DTC	147.60±89.94	149.33±73.57	p=0.75	rho=-0.38; p= 0.01
GSTRD-L-DTC	Gait Stride Length DTC	539.24±433.43	588.89±215.60	p=0.16	rho=-0.28; p= 0.06
GSTRD-H-DTC	Gait Stride Height DTC	9282.50±3614.97	11192.56±3584.04	p=0.10	rho=-0.28; p= 0.06
GSTRD-H-SD-DTC	Gait Stride Height SD DTC	6393.24±5322.10	5572.40±2738.63	p=0.79	rho=0.06; p= 0.71
GSTRD-T-DTC	Gait Stride Time DTC	81.68±41.64	61.58±28.00	p=0.10	rho=0.31; p= 0.04
GSTRD-T-SD-DTC	Gait Stride Time SD DTC	281.08±321.27	197.16±238.12	p=0.44	rho=0.27; p= 0.08
GSWT-DTC	Gait Swing Time DTC	-91.18±7.08	-93.14±6.16	p=0.54	rho=0.17; p= 0.28
GSWT-SD-DTC	Gait Swing Time SD DTC	Inf±NaN	7944.71±4482.67	p=0.73	rho=0.05; p= 0.73
GSTT-DTC	Gait Stance Time DTC	-65.09±50.46	-83.55±22.75	p=0.22	rho=-0.07; p= 0.65
GSTT-SD-DTC	Gait Stance Time SD DTC	3063.10±2299.09	1906.18±1361.25	p=0.04	rho=0.17; p= 0.26
GRS-DTC	Gait Relative Stance DTC	-99.87±0.16	-99.85±0.26	p=0.29	rho=-0.10; p= 0.52
GEXC-DTC	Gait Excursion DTC	-98.82±0.72	-99.00±0.25	p=0.94	rho=-0.13; p= 0.40
GEXC-SD-DTC	Gait Excursion SD DTC	-92.74±13.16	-97.04±2.88	p=0.70	rho=-0.17; p= 0.27
GLAT-DTC	Gait Latency DTC	1562.00±313.94	1615.08±320.74	p=0.58	rho=0.08; p= 0.63

Table S 2: Parameters definitions, means, capability in distinguishing between CNA and MCI, and correlation to MMSE score.
